# Biophysical and computational methods to analyze amino acid interaction networks in proteins

**DOI:** 10.1016/j.csbj.2016.06.002

**Published:** 2016-06-22

**Authors:** Kathleen F. O'Rourke, Scott D. Gorman, David D. Boehr

**Affiliations:** Department of Chemistry, The Pennsylvania State University, University Park, PA 16802, USA

**Keywords:** Amino acid interaction network, Allostery, Graph theory, CONTACT, Molecular dynamics, Elastic network, NMR, Coevolution, Statistical coupling analysis

## Abstract

Globular proteins are held together by interacting networks of amino acid residues. A number of different structural and computational methods have been developed to interrogate these amino acid networks. In this review, we describe some of these methods, including analyses of X-ray crystallographic data and structures, computer simulations, NMR data, and covariation among protein sequences, and indicate the critical insights that such methods provide into protein function. This information can be leveraged towards the design of new allosteric drugs, and the engineering of new protein function and protein regulation strategies.

## 1 Introduction

**Fig. 1:**
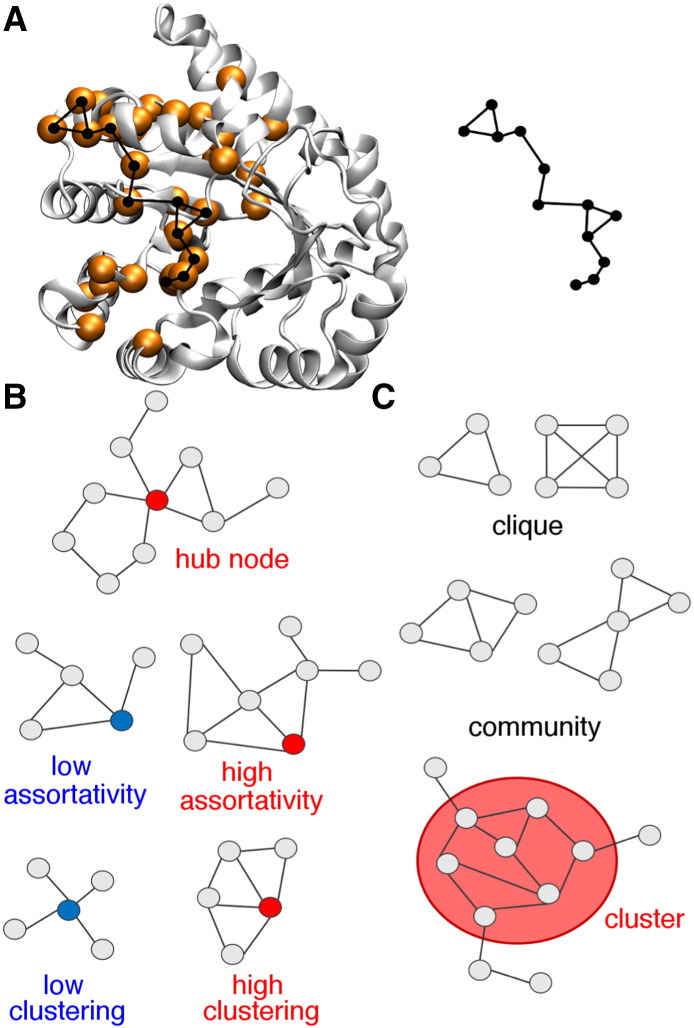
Proteins can be viewed as interacting networks of amino acid residues. A. Partial network in the alpha subunit of tryptophan synthase (PDB 1K3U) identified by NMR methods [93]. In the network representation, the nodes are the amino acid residues and represented by circles, and the edges are interactions between the residues and are indicated by lines joining the circles. B. Concepts related to network theory, including hub residues, assortativity and clustering. C. Networks can follow a hierarchy of connectivities, ranging from smaller cliques to larger clusters. Panels B and C were adapted from ref. [38].

It has long been understood that interactions at the local level (e.g. H-bonding, steric interactions) dictate the formation of protein structural elements, such as α-helices and β-sheets, and that local interactions also dictate the packing of these various structural elements to form three-dimensional protein structure (e.g. ref. [1], [2]). There is also now a better appreciation for the local interactions that are important for loop structure and dynamics (e.g. ref. [3]). With these energetic considerations in mind, globular proteins can be viewed as being held together by a series of local interactions through networks of interacting amino acid residues. These amino acid networks (Fig. 1) have also been termed ‘residue interaction networks’ [4], ‘protein structure networks’ [5], ‘contact networks’ [6], ‘pathways’ [7], ‘circuits’ [8], ‘wiring diagrams’ [9], ‘protein sectors’ [10] and so on. Intrinsic to this viewpoint is the idea that some interactions and amino acid residues are more important than others, such that the amino acid network generally represents a subset of all potential interactions and residues within a protein. In some cases, there may be multiple amino acid networks identified (e.g. ref. [11]), where local changes primarily affect the interactions between the amino acid residues involved in a particular network.

A variety of diverse structural and computational methods have been developed to delineate amino acid networks in proteins, and these methods have provided tremendous insights into protein function. In this Review, we highlight some of the different computational and experimental methods that have been used to delineate amino acid networks in proteins (Table 1), and indicate the insight that these approaches have given regarding protein function. We note that other recent review articles have been written on many of these methods, including graph theory [6], molecular dynamics (MD) simulations [12], elastic network models (ENM [13]), NMR methods to study allostery and amino acid networks [14] and bioinformatics methods to identify co-evolving residues [15], and as such, we do not treat these methods comprehensively. We also recognize that the length of this review prevents us from being exhaustive with our examples.

## 2 Network approaches to understanding protein function

In biology, network interactions have been analyzed from the species to the molecular level [16], [17], [18]. The elegance of this mathematical theory is to simplify a complex problem into a set of nodes and edges, together known as a ‘graph’ [19], [20], [21]. Graphical approaches have provided intuitive pictures and useful insights for analyzing many complex biological problems, including enzyme-catalyzed reactions [22], [23], [24], inhibition of HIV-1 reverse transcriptase [25], inhibition kinetics of processive nucleic acid polymerases and nucleases [26], protein folding kinetics [27] and drug metabolism systems [28]. In the context of protein structure, the amino acid side-chains, or whole amino acid residues, are most commonly treated as the nodes. An edge represents some type of interaction between two nodes. Edges can have a range of definitions such as the calculated energy of interaction, evolutionary conservation, or surface overlap [29], [30], [31], [32]. An important feature of edges is the weighting, which may allocate different strengths to different types of interactions and/or provide a particular cut-off distance for residues in close sequence space [33]. There are many algorithms available to construct and analyze amino acid networks using graph theory, including CSU software [34], xPyder [35], PSN-Ensemble [36] and NetworkAnalyzer [37].

Other parameters of the protein graph may be used to further analyze the network, and be related to different structural and functional properties of the protein. For example, a ‘hub node’ has a higher number of edges connected to it than other nodes [38] (Fig. 1). Residues corresponding to hub nodes may be key factors for maintaining structure and determining function. For example, a large experimental set of T4 lysozyme protein variants was studied, where some amino acid substitutions had little to no effect on the function of the enzyme and some substitutions inactivated the protein [39]. All of the deleterious substitutions were later identified as central hub nodes [40].

Connectivity is an important feature of a protein graph. The clustering coefficient, C_v_, provides a measure of connectivity through Eq. (1):

(1) $$C_{v}=\frac{2e_{v}}{k_{v}\left(k_{v}-1\right)}$$

where k_v_ is the number of neighbors to node v, and e_v_ is the number of connected pairs among v neighbors. Residues that have a high connectivity are typically linked to separate clusters or communities of residues [38]. The assortativity matrix is another parameter that helps determine the impact a node has on the network (Fig. 1). This matrix is a measure of the number of connections between nodes. A more ‘resilient’ network [41] would have a higher assortativity, providing multiple paths to connect distant regions of the protein.

A group of nodes can be classified into different types according to how a signal might be transmitted through them (Fig. 1). A clique (or k-clique) is a complete subgraph, meaning that it is a set of nodes and edges that are connected to every other node in the subgraph [38]. Similar to cliques are communities, which represent a set of connected cliques [38]. Inspection of the cliques and communities in a given protein might be used to track small ligand-induced conformational changes and signal transmission, which can be indicative of the interaction strength of the effector molecule and the quality of the network as a whole. For example, differences in the cliques and communities between the apo and ligand bound states of methionyl t-RNA synthetase were used to understand inter-domain signaling [42]. The binding of ATP induces the formation of new cliques that allow for communication between distal areas of the enzyme.

A cluster has more relaxed requirements than a clique or a community (Fig. 1). In a cluster, the nodes have a higher connectivity with each other than with nodes outside the group, but not all interact pairwise [38]. The largest cluster may be important in defining the core of the protein and can involve up to 80% of the nodes in the entire network. For example, identification of hydrophobic subclusters was used to understand long-range interactions important for stabilizing the tertiary fold of proteins [43]. In this study, it was found that the clusters were larger in thermophilic proteins, which may lead to higher temperature stability.

Measures of residue centrality, including closeness (*C*_*n*_) and betweenness, are often used to predict residues important for the transmission of information across a protein structure. The closeness centrality [44] is defined according to Eq. (2):

(2) $$C_{n}=\frac{j-1}{\sum_{i\neq n}\mathrm{sd}\left(i,n\right)}$$

where *sd*(*i*,*n*) is the shortest path between nodes *i* and *n*, and *j* is the number of nodes in the network. The betweenness centrality *B* is determined as the fraction of shortest paths that pass through a node [45]. Residues with high *C*_*n*_ or *B* have been shown to play critical roles in protein function [40], [46], [47]. Other measures of residue centrality have been proposed (e.g. ref. [48], [49] and references therein). Recent examples using these types of approaches include studies analyzing allosteric pathways in tRNA synthetases [50], G-protein coupled receptors [51], Hsp90 [52] and cyclophilin A [53].

## 3 More sophisticated structure-based approaches to network analysis

Conformational fluctuations in proteins are important in mediating their biological functions. For example, *E*. *coli* dihydrofolate reductase (DHFR) must pass through multiple conformations as it proceeds through its catalytic cycle [54]. Smaller fluctuations, such as those in side chains, may be evident in X-ray diffraction data [55], though they may be ignored during the refinement process when producing a structural model. The qFit algorithm was developed to fit these alternative side-chain conformations into the electron density derived from diffraction data [56]. The qFit algorithm is especially useful for analyzing high quality diffraction data that was collected at ambient temperature, where more substantial protein motions within the crystal would be expected [57]. These alternate side-chain conformations may lead to different interactions, providing additional information in crafting a network.

**Fig. 2:**
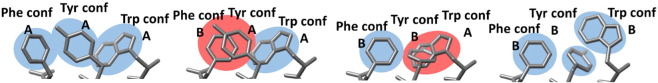
Alternate side-chain conformations using the CONTACT algorithm can be used to generate a network. For example, when the Phe sidechain transitions to conformation B there is a clash in the van der Waals radii with the Tyr sidechain. This clash is alleviated by switching Tyr to conformation B, which induces another clash which is then relieved by changing the Trp conformation. This process is repeated until there are no more steric clashes. This figure was adapted from Ref. [58].

The CONTACT algorithm has been developed to analyze potential effects of alternative side-chain conformations [58]. For the networks identified by CONTACT, nodes are defined as the side-chain and edges are defined as the steric clash of van der Waals radii between residues with alternate conformations. In the CONTACT algorithm, once it has been determined that there is a steric clash present between residues *i* and *j*, the algorithm switches residue *j* to one of its alternative conformations (Fig. 2). If this change results in another clash with some residue *k* then the algorithm moves *k* to a different conformation. This process repeats until all steric clashes are relieved, thereby identifying a particular pathway. CONTACT is capable of identifying a variety of different paths in a single network, so the edges are weighted by the number of pathways in which residues *i* and *j* are predicted to clash. This method was used to generate networks for cyclophilin A [59] and *E*. *coli* DHFR [58]; amino acid substitutions at CONTACT-determined network positions led to dramatic decreases in enzyme activity in both of these systems.

## 4 The importance of internal motions to amino acid networks

The network approaches described above primarily focus on a limited set of protein conformations. It is now recognized that rarely visited conformations can have important effects on protein function [60]. Such lowly populated conformations would have different sets of noncovalent interactions, and thus, potentially different amino acid networks. Computer simulations offer a means to predict and analyze these different amino acid networks. Such simulations can be categorized into molecular dynamics (MD) simulations and more coarse-grained models.

### 4.1 MD simulations

It has been recognized for decades that protein structure is dynamic [61], [62]. Low frequency, collective motions have been shown to exist in proteins and nucleic acids [63], [64], [65], and these motions can be important for a variety of protein functions, including switching between active and inactive states [66], cooperative effects [67], allosteric transitions [68] and assembly of microtubules [69]. In view of these important properties of proteins, it is imperative to consider not only static structural information but also the internal motions of proteins; MD simulations offer one way to do this.

MD simulations can provide trajectories of atoms or residues within a protein. One common approach to analyze these trajectories is to quantify correlated or anti-correlated motions using the dynamic cross-correlation map [70]; such motions may provide information about more concerted motions important for protein function. For example, Moustafa et al. [71] showed that the G64S substitution in the poliovirus RNA-dependent RNA polymerase leads to a higher fidelity polymerase [72], perhaps by changing (anti)correlated motions in conserved regions of the enzyme.

Non-equilibrium perturbation methods compatible with MD have also provided insight into how signals might be propagated through a protein. These methods include anisotropic thermal diffusion (ATD) [73], pump-probe molecular dynamics (PPMD) [74], and rigid residue scan (RRS) [75]. ATD is a method that examines the transfer of thermal energy through non-bonding interactions, primarily through van der Waals interactions between sidechains, by cooling the system to 10 K and applying thermal energy to a residue known to be important for function [73], [75]. PPMD reveals networks by applying an oscillating motion of varying frequency to an α-carbon, or more atoms at a greater computational cost, and identifying residues that couple with this perturbation over a nanosecond timescale [74]. An advantage of the PPMD method is that it can be applied to any standard MD simulation and is compatible with most force fields [74]. RRS creates networks by treating specific residues as rigid bodies, and analyzes how this treatment affects the conformational properties of the protein [75].

**Fig. 3:**
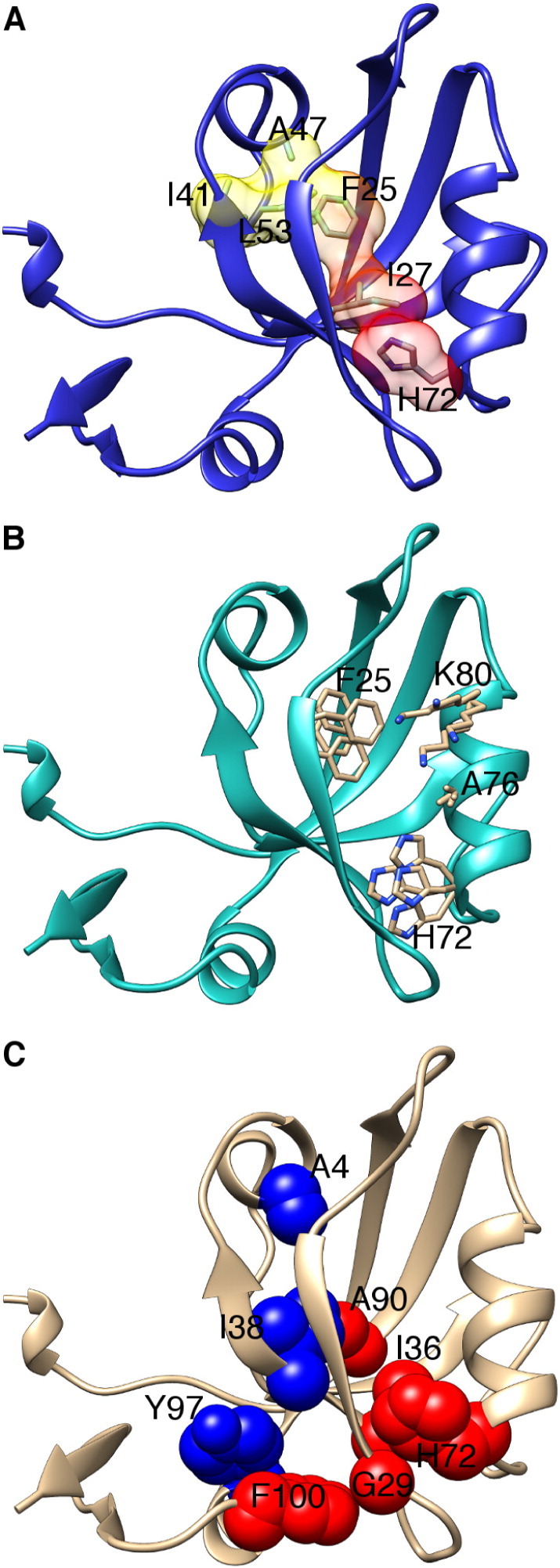
Nonequilibrium perturbations methods have been used to study amino acid networks in the PDZ domain. A. Anisotropic thermal diffusion simulations start by cooling the system to 10 K, followed by the application of a 300 K heat bath to a residue of interest. In this case, His72, an active site residue, was heated and thermal energy was diffused through van der Waals interactions Ile27, Phe25, Ile41, Ala47, and Leu53 [73] B. Pump probe molecular dynamics consist of the application of oscillating motions at varying frequencies and directions to an atom or set of atoms and observing the transfer of that motion to other residues. The oscillations at His72 transferred to Ala76, Lys80, and Phe25. Different oscillation frequencies and directions tend to show coupling to different residues, and the above representation does not represent all coupling interactions observed [74]. C. Rigid residue scan was used to systemically apply rigid body constraints to each residue in the PDZ domain to simulate point mutations [75]. By utilizing heat maps to look at the difference between the ligand-bound and unbound state of the domain during a simulation with SHAPE constraints on each residue, some residues were identified as switches (blue, including residues Ile8, Ala47, and Tyr97) while other important residues were designated as wires (red, residues Gly29, Ile36, His72, Ala90, and Phe100) depending on the degree to which they effect the transition between the bound and unbound state. Panels A, B and C were adapted from references [73], [74], [75], using PDB 1BE9.

These methods were applied to understanding the allosteric transition of the PDZ3 protein [73], [74], [75] (Fig. 3). RRS identified a network of nine residues that was deemed important for energy propagation through the PDZ3 domain [75]. Switch residues were identified as those that when rigidified in the simulation disrupted the conformational change, whereas residues that had smaller effects on the conformational change were designated as wires [75] (Fig. 3).

### 4.2 Elastic network models

**Fig. 4:**
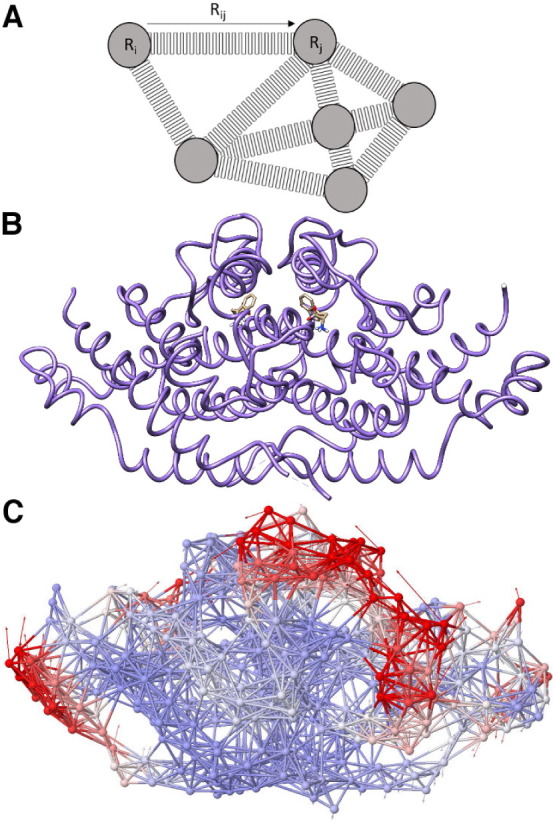
The elastic network model simulates global harmonic motions by simplifying biomolecules to a network of interacting nodes. A. In these types of models, R_i_ and R_j_ are interacting residues, and their interactions create a separation vector that is represented by R_ij_. The separation vector can be essentially treated as a ‘spring’ with a given spring constant, depending on the interaction energy. The yeast chorismate mutase homodimer (PDB 1CSM) with trypophan bound is shown as: B. a ribbon diagram, and C. a network representation with the help of ANM 2.0, a web-based tool for ENM normal mode simulations [79]. The network represention shown is ‘mode 16’, with the arrows indicating the direction of motion. ENM typically treats each alpha carbon as a node in a network of springs and nodes. The springs represent interactions within a set cutoff distance.

Course-grain simulations often employ normal mode analysis (NMA), which simulates the harmonic motions of a system. When performed on proteins, the lower frequency modes provide information about potential concerted, global motions. Early NMA was limited to smaller systems due to the computational cost of simulating every interaction between every atom. More modern applications of NMA typically take advantage of a coarse graining technique known as the elastic network model (ENM), which has allowed for the analysis of large macromolecular complexes such as the GroEL tetradecamer [76], [77], [78]. ENM simulations have such low hardware requirements compared to MD simulations that they can be simulated using web-based tools such as ANM 2.0 [79] (e.g. see Fig. 4). Modifications and improvements to ENM include the Gaussian network model (GNM) [80], the anisotropic network method (ANM) [81], the robust elastic network model (RENM) and the heterogeneous anisotropic elastic network model (HANM) [82], [83].

As an example, NMA was applied for an ENM-based simulation of the maltose transporter MalFGK2 [84]. Three low-frequency modes that contribute to channel-gating motions were analyzed, and residues involved in these low-frequency modes were perturbed by changing the spring constant between the residue and surrounding residues. The perturbations that created the greatest change in normal mode frequencies were mapped onto the protein, and the identified residues formed a network that was found to connect the channel gate on the periplasmic side of the complex, the cytoplasmic side, and the interaction points of the subunits of the complex [11].

A similar perturbation method, fluctuation perturbation analysis (FPA), was applied to a variety of proteins by Zheng et al. [85]. For example, FPA was performed to predict key residues involved in the conformational change of myosin during its power stroke, which is a necessary part of muscle contraction. The residues formed a 3D network connecting the active site to the force-generating component and the actin binding cleft [85].

## 5 NMR perturbation methods

NMR studies provide insight into the conformational dynamics of proteins over a wide-range of timescales [86]. One way to gain information about potential networks is to perturb the protein in some way (e.g. ligand binding, amino acid substitutions) and analyze correlated changes to NMR parameters. For example, Clarkson et al. generated a series of Val to Ala substitutions in eglin c and mapped dynamic responses by NMR experiments across different timescales to identify networks that propagate primarily through structural or dynamic changes [87]. The same research group performed a similar analysis with the PDZ domain [88], whose results were consistent with the MD methods discussed above.

Correlations between chemical shift changes induced by a series of perturbations can also help define a network [31], [89]. Perturbations of the protein conformational equilibrium through ligand binding or amino acid substitutions can result in chemical shift changes both local and remote from the site(s) of perturbation. Residues with covarying chemical shift changes across a series of perturbations are proposed to be involved in the same conformational change and thus belong to the same amino acid network [31]. The original method was developed by Melacini and colleagues [31], and was termed CHESCA (CHEmical Shift Covariance Analysis). Melacini and coworkers used their algorithm to define two different amino acid networks in the protein EPAC; one of these networks was associated with ligand binding and the other network was thought to be important for allosteric signaling [31]. Other groups have developed similar algorithms (e.g. Ref. [90], [91]).

**Fig. 5:**
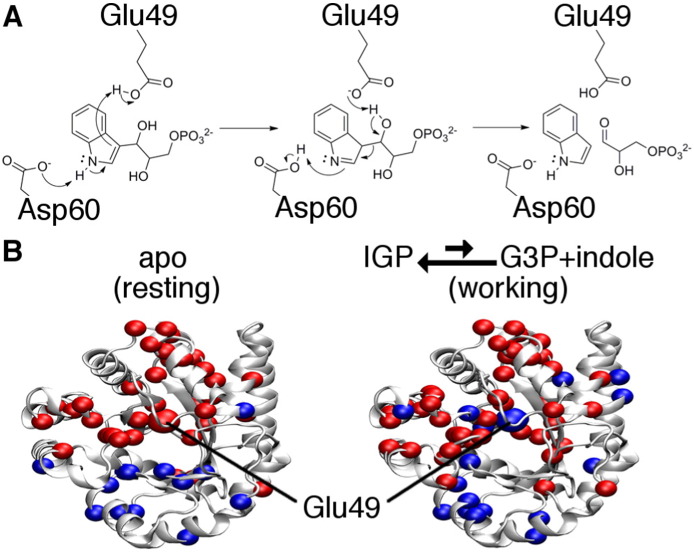
Amino acid networks in the alpha subunit of *E*. *coli* tryptophan synthase (αTS) are dependent on what is bound to the enzyme. A. Conversion of indole-3-glycerol phosphate (IGP) to glyceraldehyde-3-phosphate (G3P) and indole catalyzed by αTS, highlighting the roles of Glu49 and Asp60 in the chemical mechanism. B. The NMR method CHESCA [31] was used to delineate amino acid networks in both the ‘resting state’ in the absence of ligands (left) and ‘working state’ during active turnover [93]. In both cases, two clusters, represented by red and blue spheres, were identified. Intriguingly, the catalytic residue Glu49 changes clusters from the resting to working states, implying that these clusters might be important in regulating the catalytic activity of αTS. The protein structure is derived from PDB 1K3U.

In our own work, we have used a method similar to CHESCA to define amino acid networks in the alpha subunit of tryptophan synthase [92], [93] (Fig. 5). In our approach, we used a series of amino acid substitutions as our source of perturbations, which allowed us to interrogate amino acid networks in the ligand-free protein (i.e. the ‘resting’ state) and when the enzyme was actively turning over (i.e. the ‘working’ state) [93]. Remarkably, the amino acid networks were different between the resting and working states [93]. Of special importance was the behavior of the catalytically-important residue Glu49, which was allocated to different clusters in the resting versus working states [93]. Previous work had also shown that Glu49 undergoes an important conformational change upon binding substrate [94]; the change in network interactions may help to re-position Glu49 for catalysis.

## 6 Bioinformatic approaches to delineating amino acid networks

Since the amino acid networks are functionally-important then they may also be evolutionarily conserved. Insight into amino acid networks might then be provided by analysis of multiple sequence alignments (MSA) in the absence of structural information, as has been done through algorithms including statistical coupling analysis (SCA [10], [21], [95]), mutual information (MI [96]), McLachlan Based Substitution Correlation (McBASC [97]) and Observed Minus Expected Square (OMES [98]). A more thorough evaluation of these methods is found in Livesay et al. (2012) [15].

The SCA method has been successfully used to identify functionally significant networks in the PDZ domain [99], plant peroxidase [100], Fe/Mn superoxide dismutase [101], G protein-coupled receptor, chymotrypsin class serine protease, and hemoglobin families [102]. In SCA, energetic connectivity between amino acid residues is taken as being evolutionarily conserved to bestow common functions within protein families [99]. A key assumption of the SCA method is that the probability of finding a particular residue at a particular position without any evolutionary constraints on that position will be the same as the mean abundance of that residue in all proteins [99]; deviation from that mean frequency indicates conservation. The co-conservation of residues at two or more positions in the MSA indicates statistical coupling [99]. The statistical degree to which two sites are coupled is measured by the frequency that a change in the amino acid identity at one site is associated with a change in the coupled site [99]. The magnitude of the evolutionary constraint on a particular position is described through a Boltzmann distribution [99].

One particular noteworthy example of the power of the SCA approach is found in the engineering of a light-responsive dihydrofolate reductase (DHFR) enzyme. Ranganathan et al. used SCA on an MSA of 418 sequences to provide a basis for the discovery of novel allostery in DHFR [103]. SCA revealed a ‘sector’ that acts as a wire, which transmits allosteric signals between surface residues and the active site. To further investigate how these wires might affect DHFR activity, a light-sensitive PAS domain, LOV2, was inserted into the DHFR sequence at solvent-exposed sites. This library of DHFR-LOV2 chimeras was characterized through kinetic studies, and it was found that the chimeras with the LOV2 domain inserted at sites that were part of the cluster had an altered catalytic efficiency as compared to those that had the LOV2 domain inserted at non-cluster sites [103].

## 7 Summary and outlook

The characterization of amino acid networks by diverse experimental and computational methods has provided deeper understanding of the function and regulation of proteins. Network analyses have provided insight into internal pathways that may be important for propagating allosteric signals, and have suggested that all proteins have intrinsic allosteric properties [104]. Such properties would help to explain how amino acid changes remote from a ligand binding-site or enzyme active site [105] can nonetheless influence the function of the protein. This view has practical consequences, including in understanding drug resistance (e.g. ref. [106]), the development of new allosteric drugs that may target surface-exposed network residues [107], [108], and in protein engineering applications [109], [110], where such proteins would have wide ranging applications from biosensing to synthetic biology. The integration of various methods (e.g. ref. [111]) will likely provide greater insight into amino acid networks and provide additional lessons regarding the strengths and weaknesses of various approaches. These approaches will continue to increase our understanding of protein function, and provide novel avenues towards modulating these functions in practical applications of drug and protein design.

## Figures and Tables

**Table 1:** Brief summary of the computational and biophysical methods to analyze amino acid networks in proteins addressed in this review.

	Brief description	Comments
Graph theory	Formulized math-based approach to identify residue connectivities and potential allosteric paths in proteins.	Often used with static protein structures, which may not reveal the full range of potential contacts in a dynamic protein. Combining graph theory and dynamic information (e.g. through MD simulations) is a powerful approach.
CONTACT	Identify alternative side-chain conformations and their contacts based on high quality X-ray diffraction data and electron density maps.	High quality diffraction data is generally required, especially at non-cryogenic temperatures. This method can be combined with principles from graph theory.
MD simulations and elastic network approaches	Computer simulations and calculations of internal protein motion over a wide range of timescales.	These computer simulations offer trajectories of motions, often missing in biophysical approaches that study protein structural dynamics. These methods are often combined with principles from graph theory. Non-equilibrium approaches offer additional ways to identify potential allosteric pathways. These approaches become more convincing with experimental validation.
Analysis of amino acid perturbations using NMR	Perturbations of protein structure/dynamic (e.g. through amino acid substitutions) can be followed on an atom-specific basis to reveal potential allosteric pathways.	NMR offers great experimental methods to analyze internal motions of proteins over a wide range of timescales at atomic resolution. These methods are often used to validate MD simulations, and/or extend analysis to longer timescales (i.e. > microseconds). Solution-state NMR may be limited to smaller proteins (i.e. < 100 kDa).
Amino acid sequence based analysis of networks	In an amino acid sequence alignment of hundreds to thousands of similar proteins, identify residue positions that covary, implying coevolution.	An appropriate number of protein sequences and an appropriate level of sequence diversity are required. The information may be difficult to understand in the absence of a structural/dynamic rationale.
